# Cardiovascular Disease Mortality Attributable to Low Whole-Grain Intake in CHINA: An Age-Period-Cohort and Joinpoint Analysis

**DOI:** 10.3390/ijerph19127096

**Published:** 2022-06-09

**Authors:** Fangyao Chen, Yuxiang Zhang, Shiyu Chen, Aima Si, Weiwei Hu

**Affiliations:** 1 Department of Epidemiology and Biostatistics, School of Public Health, Xi’an Jiaotong University Health Science Center, No. 76 Yanta Xilu Road, Xi’an 710061, China; yuxiang_zhang@stu.xjtu.edu.cn (Y.Z.); shiyu_chen@stu.xjtu.edu.cn (S.C.); siaima@stu.xjtu.edu.cn (A.S.); xjhww2016@stu.xjtu.edu.cn (W.H.); 2 Department of Radiology, First Affiliated Hospital of Xi’an Jiaotong University, No. 277, Yanta Xilu Road, Yanta District, Xi’an 710061, China

**Keywords:** cardiovascular disease, attributive mortality, low whole-grain intake, age-period-cohort analysis

## Abstract

Cardiovascular disease (CVD) is the leading cause of death worldwide. Low whole-grain intake is found to be one of the most important risk factors for cardiovascular disease development and progression. In this study, we focused on exploring the long-term trends of low whole-grain intake attributed to cardiovascular disease mortality in China during 1990–2019 and relative gender differences. Study data were obtained from the Global Burden of Disease (GBD) 2019 study. We used the age-period-cohort model to estimate the adjusted effect of age, period, and cohorts. Annual and average annual percentage changes were estimated by joinpoint regression analysis. We observed an increasing trend with a net drift of 1.208% for males and 0.483% for males per year. The longitudinal age curve suggested that the attributed rate increased for both genders. Period and cohort effects all suggested that the risk for males showed an increased trend that was higher than that of females. Our findings suggest that males and senior-aged people were at a higher risk of cardiovascular disease mortality attributed to low whole-grain intake. Effective strategies are needed to enhance people’s health consciousness, and increasing whole-grain intake may achieve a better preventive effect for cardiovascular disease.

## 1. Introduction

Cardiovascular disease (CVD) is defined as a group of disorders involving the circulatory system. It is the leading cause of death globally [1,2], as well as in China [3]. By modeling CVD from 2010 to 2030, Moran et al. predicted that the incidence of CVD in China will continue to rise, and its disease burden will remain at a high level [4]. With the deepening of aging, CVD has become a serious public health problem in China and globally. It is urgent to curb the growing trend of its incidence and reduce its impact on general health.

Eating patterns are defined as the frequency, amount, variety, or combination of different types of food consumed in the daily diet which influences human health [5]. A published study suggested that diet pattern has a potential influence on CVD initiation and progression [6]. Targeted dietary and behavioral interventions, preventive advice, and comprehensive interventions for people with high CVD risk have become the focus of attention in the field of CVD research [6]. A healthy dietary pattern is highly beneficial to one’s physical well-being; however, survey data shows that the food and nutrient intake of people in 195 major countries and regions around the world does not meet the recommended pattern by the World Health Organization (WHO) [7,8]. Furthermore, among the 15 bad eating habits, the highest mortality was caused by a high-sodium (salt) diet, insufficient intake of whole grains (WGs), and fruit [7,8].

Grains are a general term for grain crops which are the key basic elements of a daily diet and the source of multi-nutrients, including B vitamins, minerals, and dietary fiber [9]. Depending on the processing degree, grains or grain products can be classified into WGs and refined grains (RGs) [10]. WGs are whole, milled, broken, or flaked grains whose basic components include starchy endosperm, germ, and cortex; this is in contrast to RGs, which retain only the endosperm parts. Common WGs include brown rice, whole wheat, corn, beans, etc., whereas RGs usually include white flour, white rice, white bread, degermed cornflower, and others [10].

The Chinese Dietary Guidelines published by the Chinese Nutrition Society has recommended eating 50 to 150 g of WGs and mixed beans every day for Chinese adults, which is equivalent to 1/3 to 1/4 of all grain intake in a day [11]. The intake of WGs has long been acknowledged to be associated with a low risk of several important chronic diseases including CVD [12]. However, a survey study indicated that more than 80% of the adults in China have a seriously insufficient intake of WGs [13].

Most of the previously published studies in China focused on the association between CVD and the intake of fruit, vegetables, and meat, as well as other sources of nutrition; however, WGs have not been specifically studied much. Besides, the long-term trends of CVD mortality attributable to low WG intake across age and gender in China have not been comprehensively investigated. Dietary patterns vary across different social and cultural backgrounds, and their association with the particular disease are also influenced by sociocultural factors. Therefore, in this study, we focused on the investigation of the effect of age, period, and cohort on low WG intake attributed to CVD mortality from 1990 to 2019 in China and explore the gender difference. This study was conducted based on the public data from the Global Burden of Disease (GBD) 2019 study. Our findings provide quantified results and evidence for the prevention of CVD.

## 2. Materials and Methods

### 2.1. Research Data

Study data were obtained from the GBD 2019 study, which was a large, international cooperation project directed by the Institute for Health Metrics and Evaluation [1,14]. The GBD 2019 study provided comparable and comprehensive measurements of a collaboration of cause-specific mortality for 369 diseases and 87 risk factors across 204 countries [1,14]. The data can be used, shared, modified, or built on via the Open Data Commons Attribution License [15] through the GBD Data Tool repository (http://ghdx.healthdata.org/gbd-results-tool, accessed on 28 January 2022).

### 2.2. Measurements

In the GBD 2019 study, the data on CVD mortality in China were collected from the Disease Surveillance Points System, the Maternal and Child Surveillance System, and the Chinese Center for Disease Control and Prevention’s Cause-of-Death Reporting System in China [1]. CVD morbidity and mortality were diagnosed and defined based on the World Health Organization’s clinical criteria and the International Statistical Classification of Diseases and Related Health Problems, 9th and 10th revisions [1].

The original data for estimating low WG intake in China were collected from the China National Nutrition Survey (CNNS) program, and other published studies [14,16]. The CNNS program collected dietary data (as well as other health-related data) at a household level using interviews [16]. The samples for the surveys were selected to represent the population structure of all ages in China and covered 31 provinces (including autonomous regions and centrally administered municipalities) in China, ensuring good representativeness [16]. In the GBD data, the low WG intake was defined as WG intake less than 140 to 160 g of WGs or WG products (germ, endosperm, and bran in natural proportion) from biscuits, rice, bread, and other sources [16,17].

The main measurements used in this study include gender, the crude mortality rate (CMR), and age-standardized mortality rate (ASMR). The CMR and ASMR were the main outcomes in the analysis. The CVD mortality rates attributed to low WG intake were estimated using the population-attributable fractions (PAF) for each gender, year, and age group, where the estimated attributable mortality rates were equal to the mortality multiplied by the PAF [1].

### 2.3. Statistical Analysis

In this study, we used the joinpoint regression analysis to estimate the long-term trends of CVD mortality attributable to low WG intake from 1990 to 2019. The joinpoint regression is also called the piecewise regression, which was first proposed by Kim and colleagues in 2000 [18]. The basic idea of the joinpoint regression is to divide a long-term trend line into several segments, and each segment is described with continuous linearity [18]. In the joinpoint regression analysis, we assumed the mortality rates followed a Poisson distribution. The average percent changes (APCs), average annual percent changes (AAPCs), and the corresponding 95% confidence interval (CI) were estimated. The joinpoint regression was performed with the Joinpoint Regression Program (version 4.9.0.0, March 2021; Statistical Research and Application Branch, National Cancer Institute, Rockville, MD, USA).

The cumulative health risks were estimated through an age-period-cohort (APC) model. To conduct the APC model, it was required for the included records to be converted to successive 5-year age groups and consecutive 5-year periods. The rate ratio (RR), local drift, net drift, and their corresponding 95% CIs were estimated. The APC model was conducted with the APC Web Tool (https://analysistools.nci.nih.gov/apc/, accessed on 23 March 2022) [19].

Statistical graphs were generated with the R programming language (version 4.0.3, The R Foundation, Vienna, Austria) and RStudio software (Version 1.1.463, RStudio Inc., Boston, MA, USA). The level of statistical significance was 0.05 (two-tailed) for all analyses.

## 3. Results

### 3.1. Long-Term Trend of CMR and ASMR

**Figure 1:**
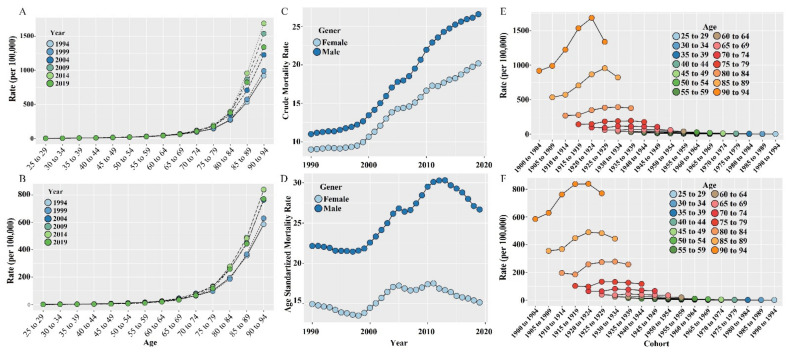
The long-term trends of low whole-grain intake attributed to CVD mortality (per 100,000) in China during 1990–2019. (**A**) Age-specific CVD mortality rates for males; (**B**) age-specific CVD mortality rates for females; (**C**) gender-specific CMR along with time periods; (**D**) gender-specific ASMR along with time periods; (**E**) age-specific CVD mortality rates along with birth cohorts for males; (**F**) age-specific CVD mortality rates along with birth cohorts for females.

Our study included 6,350,191 Chinese CVD cases (including 3,667,817 men and 2,682,374 women) aged 25 to 94 years from 1990 to 2019. The period-wise long-term trends of the CMR (per 100,000) attributed to low WG intake for males, females, and all genders are presented in Figure 1A (males) and Figure 1B (females). As shown in the figure, we could observe an increasing trend of the low WG intake attributed to CMR for males and females, as well as for the whole population; however, the CMR of females was lower than that of males. Despite gender, the peak of the CMR existed in the period of 2014 and slightly decreased after that. The CMR for different periods showed a continued exponential increase along with age groups.

As shown in Figure 1C, the CMR (per 100,000) of all ages for males was higher than that of females. Before the year 2000, the increasing trend for both genders was not significant; however, it accelerated after the year 2000 and a downward trend in growth has not yet been observed.

The long-term trend of ASMR (per 100,000) was presented in Figure 1D. As presented, the ASMR of males was higher than that of females for all time periods. Inconsistent long-term trends across periods can be observed for both genders in the CMR and ASMR.

The age-specific trends along birth cohorts were shown in Figure 1E (for males) and Figure 1F (for females). The CMR (per 100,000) in different age groups for both males and females decreased across birth cohorts. To be specific, we observed that the mortality rate in the elderly groups (75 to 79, 80 to 84, 85 to 89, and 90 to 94) increased first and then decreased, along with birth cohorts. The trends were relatively stable for other age groups.

### 3.2. Age-Period-Cohort Analysis

**Figure 2:**
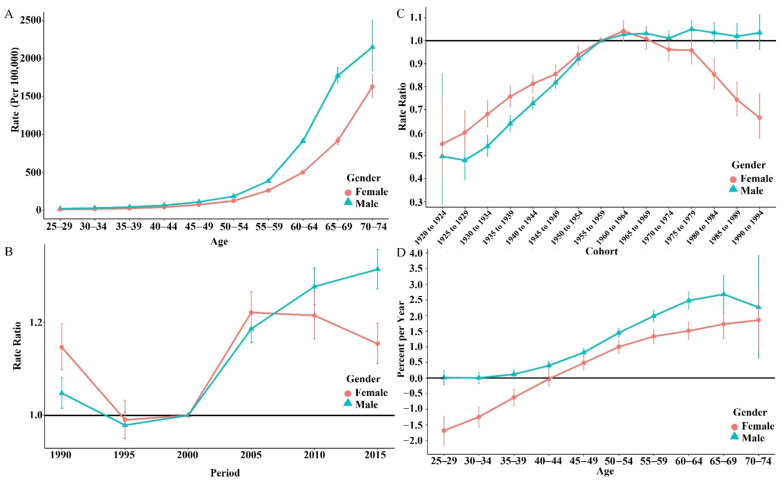
Effect of age, year period, and birth cohort and estimation of local drifts by genders. (**A**): Crude mortality rates (per 100,000) and corresponding 95% CI (the error bars) along age groups; (**B**): rate ratio (RR) and 95% CI (the error bars) for cohort effect; (**C**): RRs and 95% CI (the error bars) for period effect; (**D**): local drift (percent per year) along with age groups.

We further conducted the APC model to estimate the effect of age, period, and birth cohort on the low WG intake attributed to CVD mortality. The estimated RRs and corresponding 95% CI (presented with error bars) are presented in Figure 2.

The age effect on low WG intake attributed to CVD mortality rates (per 100,000) controlling for the time period and the birth cohort is shown in Figure 2A. The rates increased along with age, showing an exponentially increasing trend. Before the age group of 50 to 59, the rates (per 100,000) for both genders were close to each other; however, the increasing trend of males was more accelerated than that of females thereafter.

For the effect of the period controlling for age and birth cohort, an inverted S-shaped trend was observed, as shown in Figure 2B. The RRs for both genders presented a downward trend from 1990 to 1995. Between 1995 to 2000, the trends were relatively stable for both genders. The RRs of males were lower than females before the year 2005 and then became higher. The RRs increased for males relatively quickly, whereas for females, the RRs showed a gradual downward trend after the year 2005.

For the effect of birth cohorts controlling for age group and period, a U-shaped trend for females and an upward trend for males was observed, as shown in Figure 2C. The RRs for both genders crossed twice along with birth cohorts. The peak of RRs for females appeared in the birth cohort of 1960 and decreased thereafter. The RRs for males showed a stable increasing trend along with all birth cohorts. The RRs for males were slightly lower than that of females in earlier cohorts (before 1960 to 1964), whereas for birth cohorts after that, the RRs for males were higher than that of females.

For the local drifts, the peak of local drift for Chinese males (2.681, 95% CI: 2.089~3.277) appeared in the age group of 65–69, whereas that for females (1.851, 95% CI: 0.899~2.8121) appeared at 70–74 (both with *p* < 0.05) and continues to grow, as shown in Figure 2D. The overall net drift for males was 2.409% (95% CI: 2.084~2.734%; *p* < 0.001) and 0.634% (95% CI: 0.388~0.880%; *p* < 0.001) for females.

### 3.3. Joinpoint Analysis

**Figure 3:**
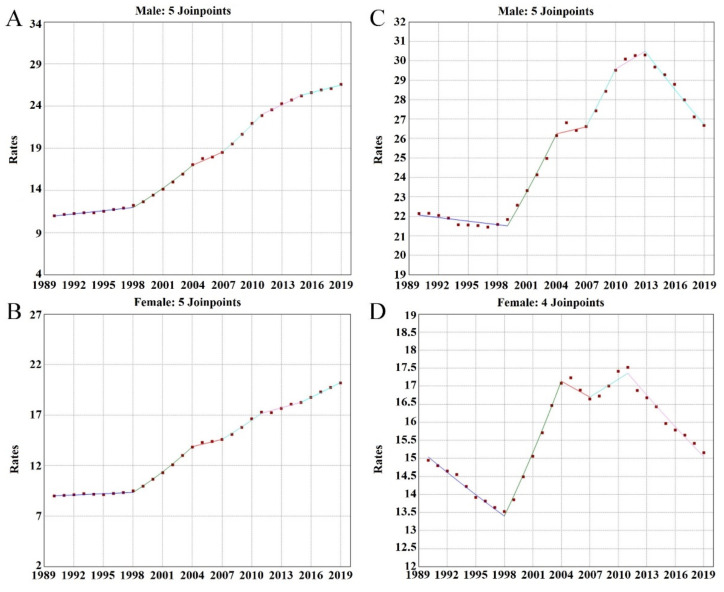
Results obtained from joinpoint analysis. (**A**) Segments of CMR for males; (**B**) segments of CMR for females; (**C**) segments of ASMR for males; (**D**) segments of ASMR for females.

To further understand the non-linear long-term trend of CMR and ASMR, we performed a joinpoint regression analysis; the estimated APCs and AAPCs are shown in Table 1 and Table 2, and the identified segments as well in Figure 3.

For the CMR, as shown in Table 1 and Figure 3A,B, the increasing trend of the CMR can be divided into six segments for both genders and the whole population. The general trends for males and females were approximately the same, as the APC for males was 3.1% and 3.0% for females from 1990 to 2019. As shown in Table 1, the increasing trend has slowed down. The peak existed in the time period from 1998 to 2004 for both males by 6.0% (95% CI: 5.6~6.3%; *p* < 0.001) per year, and for females by 6.8% (95% CI: 6.7~7.1%; *p* < 0.001) per year, then decreased gradually for both genders.

For the ASMR, as shown in Table 2 and Figure 3C,D, the increasing trend for males and females was different. For males, the ASMR continuously increased and reached its peak in the time period from 1999 to 2004 at 4.1% (95% CI: 3.5~4.6%; *p* < 0.001) per year. After that, the ASMR gradually decreased. For females, the long-term trend showed the same pattern as males, whereas the average trend of the ASMR for females fluctuated within a relatively stable range as the AAPC equals 0.0% (95% CI: −0.2~0.2%; *p* > 0.05).

## 4. Discussion

Risk factors for the development and progression of CVD have been studied in many previously published studies. Low WG intake is probably one of the most important but easiest-to-be-ignored factors. With the literature review, we found more and more researchers have noticed the impact of WGs on CVD and the association between CVD and WG intake [13,20,21,22,23,24,25]. However, few studies have focused on a comprehensive analysis of the long-term trend of CVD mortality attributable to low WG intake in China. 

Published studies have suggested that increases in WG intake by 15 g per day could reduce the overall mortality and morbidity of various chronic diseases by approximately 2% to 19% [20]. However, a survey study found that the daily intake of WGs for Chinese adults is 13.9 g, and that for women it is 14.6 g, which is far lower than the recommended intake [13].

In this study, we aimed to explore the long-term trends of low WG intake attributed to CVD mortality from 1990 to 2019 in China. We also investigated the effects of age, time period, and birth cohort across genders. Our findings suggested that males had higher CVD mortality rates attributable to low WG intake than females.

The findings of a recently published cohort study conducted in China suggested that a low WG diet is associated with a higher risk of cardiovascular events [21]. A newly published prospective study based on a long-running birth cohort study in Australia also concluded the same result [22]. Active ingredients in whole grains, such as dietary fiber, vitamins, minerals, antioxidants, lingams, phytosterols, etc., can affect cardiovascular health through the metabolism of sugar, blood lipids, lipoproteins, and vascular endothelial function [23,24,25].

Age has been regarded as an important risk factor for CVD [26]. Our findings confirmed that CVD mortality also varies across senior age groups, as shown in Figure 1A and Figure 1B. During the past few decades, the health care service in China and the health status of Chinese people have been greatly improved [27]. With the increase in average life expectancy and social development, the degree of aging in China is gradually deepening [28]. Those changes, together with transformations in lifestyles, have had an influence on the initiation and progression of CVD. 

Senior people, compared to the younger population, may eat less and have a weaker digestive function, and lower food intake may reduce senior citizens’ intake of WGs. Unlike refined grains, WGs may have a rough texture and are harder to digest. Besides, the elderly often have indigestion and chewing problems [29] which make them tend to have lower consumption of WGs than young people. This could make the intake of WGs decrease along with age, and increase the low WGs attributed to CVD mortality.

Period effects suggested that the attributed CVD CMR increased from 1990 to 2019, whereas the increasing trend has been slowing down, as the ASMR has already passed its peak and gradually decreased. The development of whole-grain foods in China started in 2011 (which is not early compared with other countries), and the current main problem is that, currently, there is a small quantity and variety of whole-grain foods and the taste is poor, making it difficult to meet the needs of consumers [30]. Traditional risk factors including high intake of red meat, processed meat, sugary drinks, and even trans fats have been well recognized by people, whereas the importance of WGs has only begun to be understood in recent years. A survey conducted in China indicated that only 24.6% of consumers can clearly tell what WGs are, whereas only 5.84% can reach the recommended intake amount [30].

The period effect is the sum of the effects of all other factors affecting all age groups during the data collection period [31,32]. It contains the effects of a range of environmental, social, and economic factors and how they have changed over the study period [31,32]. In this study, the possible reasons for the period effect also include other factors that have an impact on CVD mortality risk, such as drinking, smoking, changes in life behavior, environmental factors (e.g., air quality and temperature), and others. Although we cannot decompose and adjust the effects of these factors separately, adjustment for period effects in the APC analysis could control for the influence of those factors to a certain degree.

In this study, the effect of the birth cohort showed a descending trend and suggested that the early birth cohort suffered higher low WG intake attributed to CVD mortality. The effect of the birth cohort may be caused by the exposure to risk factors in early life. The declining trend could mainly be the result of the improvement of health care systems and living conditions in China [3].

A significant gender difference was observed in this study. A published study suggested that CVD mortality in males is higher than that of females; however, CVD acts as the first leading cause of death in women [29], which is consistent with our findings. This is partly because males are more likely to be associated with CVD risk factors such as alcohol, smoking, and red or produced meat [33]. Whereas, compared with men, women are more health conscious and usually pay more attention to collecting and obtaining health-related information, which makes them more likely to change unhealthy lifestyles [34]. Published studies suggest that, compared with men, aged females usually had a higher consumption of WGs [35,36], which may help them benefit more from the protective effect of WGs.

Based on published studies, CVD is a largely preventable disease [37]. This suggests that, theoretically, most of the occurrence of CVD could be prevented by living a healthy or low-risk lifestyle. Low WG intake is among the four highest contributors to mortality [7] and is a controllable factor. This also suggests that taking measures to enhance the health awareness of people, especially males, to live a healthier life in order to reduce CVD mortality.

In our analysis, we also used the joinpoint regression. Traditional linear models can only describe or predict one trend, and time-series models also have some limitations. As presented in Figure 1, the long-term trend of low WG intake attributed to CVD mortality was not simply linear. Therefore, dividing the long trend into several statistically significant trend segments by model fitting would be more appropriate. This is one of our strengths.

There are also some limitations in this study. The low WG intake data were estimated rather than directly measured. Though the data is reliable, potential bias may not be totally avoided. Then, due to the limitation of the data, the demographical difference (especially in dietary habits) between different regions in China was not taken into consideration. The analysis was conducted based on summarized data, whereas a cohort based on a large population may provide more detailed results.

In summary, in this current study, we explored the long-term trends of low WG intake attributed to CVD mortality from 1990 to 2019. The CMRs increased for both genders, whereas the CMR for males was higher than that of females. The ASMR reached its peak for both genders and showed a decreasing trend, although the ASMR for males is still higher than for females. APC analysis suggested that the CVD mortality attributable to low WG intake increased along with age, whereas it decreased along with birth cohorts for both genders. The risk is increasing along with the time period; however, the increasing trend has been slowed down.

In general, based on our findings, we can conclude that the CVD mortality attributable to low WG intake has not been well controlled over the past three decades. We suggest that males and senior-aged people are at high risk of CVD mortality attributable to low WG intake. Gradually increasing the intake of WGs and WG products may provide a protective effect. Our findings may provide useful information and quantified evidence for public health policymaking, and help promote healthy eating to reduce the risk of CVD mortality.

## 5. Conclusions

As a result, the crude and age-standardized mortality rates increased for both genders from 1990 to 2019, whereas the increasing trend was slowed down. A gender difference was observed. Males and senior citizens may have higher CVD mortality attributable to low WG intake. Though the increasing trend was slowed down, effective strategies still need to be taken to enhance people’s health consciousness and improve WG intake levels to achieve better prevention of cardiovascular mortality.

## Figures and Tables

**Table 1:** Annual percent change for crude mortality attributable to low whole-grain intake by gender in China, 1990–2019.

Segment		No. of Death ^#^	Year	APC (95% CI)	*t* (*p*)
Male	Trend 1	661,850	1990~1998	1.1 (0.9, 1.3)	11.7 (<0.001)
	Trend 2	596,401	1998~2004	6.0 (5.6, 6.3)	38.6 (<0.001)
	Trend 3	373,521	2004~2007	3.0 (1.8, 4.1)	5.5 (<0.001)
	Trend 4	592,942	2007~2011	5.6 (5.0, 6.1)	23.4 (<0.001)
	Trend 5	692,046	2011~2015	2.4 (1.9, 2.8)	11.9 (<0.001)
	Trend 6	751,059	2015~2019	1.2 (0.9, 1.4)	9.5 (<0.001)
	AAPC	-	-	3.1 (2.9, 3.3)	- (<0.05) *
Female	Trend 1	494,414	1990~1998	0.5 (0.3, 0.6)	5.6 (<0.001)
	Trend 2	451,587	1998~2004	6.8 (6.7, 7.1)	49.1 (<0.001)
	Trend 3	282,907	2004~2007	1.7 (0.6, 2.7)	3.5 (0.004)
	Trend 4	430,779	2007~2011	4.2 (3.7, 4.6)	18.9 (<0.001)
	Trend 5	482,611	2011~2015	1.6 (1.1, 2.0)	7.9 (<0.001)
	Trend 6	399,331	2015~2019	2.6 (2.3, 2.8)	21.4 (<0.001)
	AAPC	-	-	3.0 (2.7, 3.2)	- (<0.05) *

*: software provided significant results without exact information of test statistics and *p*-value. ^#^: number of CVD deaths attributable to low GW intake obtained from the GBD 2019 database.

**Table 2:** Annual percent change for age-standardized mortality attributable to low whole-grain intake by gender in China, 1990–2019.

Segment		No. of Death ^#^	Year	APC (95% CI)	*t* (*p*)
Male	Trend 1	746,208	1990~1999	−0.3 (−0.5, −0.1)	−3.4 (0.005)
	Trend 2	512,043	1999~2004	4.1 (3.5, 4.6)	15.4 (<0.001)
	Trend 3	373,521	2004~2007	0.4 (−1.1, 2.0)	0.6 (0.549)
	Trend 4	432,525	2007~2010	3.6 (2.0, 5.2)	5.1 (<0.001)
	Trend 5	497,638	2010~2013	1.0 (−0.4, 2.4)	1.6 (0.135)
	Trend 6	1,105,884	2013~2019	−2.2 (−2.2, −1.9)	−18.1 (<0.001)
	AAPC	-	-	0.7 (0.4, 0.9)	- (<0.05) *
Female	Trend 1	494,414	1990~1998	−1.4 (−1.6, −1.3)	−19.0 (<0.001)
	Trend 2	451,587	1998~2004	4.2 (3.9, 4.5)	28.2 (<0.001)
	Trend 3	282,907	2004~2007	−0.9 (−2.1, 0.44)	−1.5 (0.157)
	Trend 4	430,779	2007~2011	1.0 (0.4, 1.6)	3.4 (0.004)
	Trend 5	881,943	2011~2019	−1.8 (−1.9, 1.6)	−25.1 (<0.001)
	AAPC	-	-	0.0 (−0.2, 0.2)	- (>0.05) *

*: software provided significant results without exact information of test statistics and *p*-value. ^#^: number of CVD deaths attributable to low GW intake obtained from the GBD 2019 database.

## Data Availability

The data involved in this study can be obtained at the GBD 2019 data portal (http://ghdx.healthdata.org/gbd-results-tool, accessed on 28 January 2022).
